# Chromatin-remodeling factor, RSF1, controls p53-mediated transcription in apoptosis upon DNA strand breaks

**DOI:** 10.1038/s41419-018-1128-2

**Published:** 2018-10-22

**Authors:** Sunwoo Min, Keeeun Kim, Seong-Gwang Kim, Hyeseong Cho, Youngsoo Lee

**Affiliations:** 1 Genomic Instability Research Center, Ajou University School of Medicine, Suwon, 16499 Korea; 2 Department of Biochemistry & Molecular Biology, Ajou University School of Medicine, Suwon, 16499 Korea; 3 Department of Biomedical Sciences, The Graduate School of Ajou University, Suwon, 16499 Korea; 4 Genome Stability Institute, Ajou University School of Medicine, Suwon, 16499 Korea

## Abstract

Remodeling and spacing factor 1 (RSF1), which is one of chromatin-remodeling factors, has been linked to the DNA damage response (DDR) and DNA repair. However, the biological consequence of RSF1 deficiency in DDR in vivo and its molecular mechanisms remain unknown. Because defective DDR is related to neuropathological phenotypes, we developed neural-specific *Rsf1* knockout mice. *Rsf1* deficiency did not result in any neuropathological abnormalities, but prevented neural apoptosis triggered by excessive DNA strand breaks during neurogenesis. Likewise, cell death was significantly reduced in *RSF1* deficient human cell lines after DNA damage, and the global transcriptome of these cells revealed that the expressions of p53 downstream genes were significantly reduced upon DNA strand breaks. Inactivation of these genes resulted from decreased binding of p53/p300 complex and subsequent reduction of H3 acetylation at their promoters. Our data show that RSF1 is necessary for p53-dependent gene expression in response to DNA strand breaks via controlling the accessibility of p53/p300 complex to its target genes and contributes to the maintenance of cellular integrity.

## Introduction

Genomic stability is fundamental for proper development and lifelong functioning, and DNA repair pathways are crucial to counteract the various endogenous and exogenous sources of DNA damage that continuously challenge this stability. Numerous diseases are caused by genomic instability, including developmental defects and cancer^1^. As the chromatin structure in eukaryotes is a barrier to DNA damage detection and repair, chromatin-remodeling complexes are important for ensuring an appropriate DNA damage response (DDR) and repair, and eventually for maintaining genomic stability^2^.

Remodeling and spacing factor 1 (RSF1) is a chromatin-remodeling factor initially identified as a transcriptional activator with the FACT (facilitates chromatin transcription) complex. RSF1 with SNF2h ATPase forms RSF complex in the ISWI family, which can reposition the nucleosome for transcriptional regulation^3,4^. Several reports demonstrated that *RSF1* is overexpressed in multiple types of tumors and correlates with their aggressiveness in terms of tumor size and TNM (classification of malignant tumors) stages^5–13^. *RSF1* overexpression was also recently correlated with pathological type, tumor aggressiveness, and *TP53* status in breast cancer, such that the overall survival was shorter for breast cancer patients with tumors overexpressing *RSF1* and harboring *TP53* mutations^14^.

RSF1 may also protect against DNA damage, possibly by influencing ATM-dependent checkpoint signaling. We and other groups reported previously that RSF1 is required for proper phosphorylated H2AX (γ-H2AX) propagation and DNA damage repair^15–17^. Also ATM likely binds to and modifies RSF1 upon DNA damage^15,17^. ATM kinase helps maintain genomic stability in response to DNA strand breaks, and *ATM* mutations cause ataxia telangiectasia in human, which manifests clinically as defects of the immune system, increased tumor incidence, ataxia, and neurodegeneration^18^. A well-known substrate of ATM kinase is p53, which binds to the promoters of target genes and regulates target gene expressions that govern cell-cycle arrest and apoptosis in response to DNA damage^19^. Gene mutations or inactivation of p53 and defects in its downstream network lead to genomic instability and promote cancer development^20,21^.

Although RSF1 has been implicated in the DDR, the biological consequences of *RSF1* deficiency in proliferating cells under DNA damage and the molecular mechanisms for cell fate decisions remain unclear. Here, we report that RSF1 is crucial for p53-dependent cell fate determination upon DNA damage. To study the role of RSF1 in vivo, a mouse model with conditional knockout (cKO) of *Rsf1* during neurogenesis was generated. Although *Rsf1* was dispensable for brain development and function, the developing nervous system in *Rsf1* cKO mice was protected from cell death triggered by exogenously induced DNA strand breaks. This protection was confirmed in vitro by analyzing human *RSF1* deficient cell lines. Gene-expression profiling of RNA sequencing (RNAseq) from *RSF1* KO cells revealed reduced expression of p53-target genes, particularly those related to apoptosis. This reduction resulted in part from the disruption of p53/p300 binding and the consequent reduction of H3 acetylation on the promoters of p53-target genes upon DNA strand breaks. These data demonstrate that RSF1 is necessary for proper p53-dependent signaling induced by DNA strand breaks.

## Results

### *Rsf1* is dispensable for brain development

Human RSF1 is immediately recruited to sites of DNA damage^15^, and whether this is also the case with the murine RSF1 was first examined. The kinetics of murine RSF1 accumulation to the sites of DNA strand breaks induced by microirradiation was similar to that of the human RSF1 protein (Figure S1a). To study the in vivo function of RSF1, an *Rsf1* cKO animal model was generated using *Flp-FRT* and *Cre-LoxP* recombination (Figure S1b). We obtained a floxed animal model provided by the Canadian Mouse Repository at the Hospital for Sick Children, and then cross-bred with the mouse model expressing *Cre* recombinase under the control of the *Nestin* promoter (*Nes-Cre* line) to inactivate *Rsf1* selectively in the neuroprogenitor cells during development. The nervous system is a good target organ to examine DDR, since the distinct developmental stage allows us to look into difference of DDR and damage repair in proliferation, differentiation, and maturation of cells, as defective DDR and damage repair in the nervous system has severe consequences in humans^1,22^. Furthermore, dynamic changes of chromatin environment and epigenetic regulation are one of critical regulatory factors for the proper neurogenesis^23,24^. In the mouse model, exon 4 of *Rsf1* encoding a part of the WHIM domains was floxed (Figure S1b, right), and then successfully deleted in a *Nes-Cre* line (*Rsf1*^*Nes-Cre*^ as indicated in figures) (Figure S1c), with no RSF1 protein detected throughout the central nervous system (Figure S1d).

**Fig. 1:**
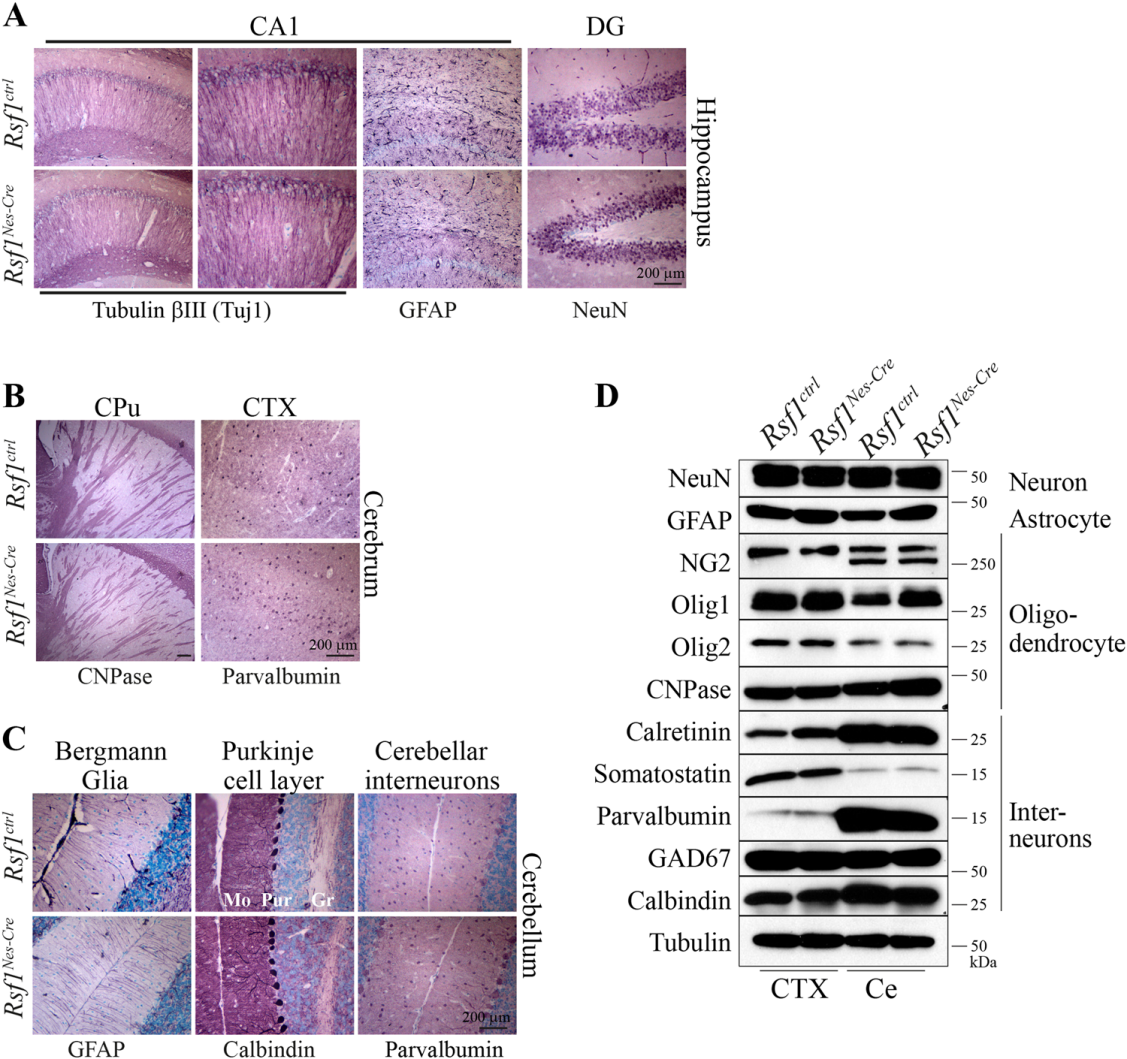
*Rsf1* cKO brain develops normally. **a**, **b**. Immunohistochemical analysis of adult brains (6-month old) with markers for neurons (Tuj1 and NeuN) and astrocytes (GFAP) (**a**) and oligodendrocytes (CNPase) and parvalbumin positive interneurons (**b**). There were no neuroanatomical differences between *Rsf1*^*ctrl*^ and *Rsf1*^*Nes-Cre*^ brains with regard to the hippocampus (CA1, cornu ammonis area 1; DG, dentate gyrus), caudate putamen (CPu), or cerebral cortex (CTX). **c** Immunohistochemical analysis of Bergmann glia (GFAP), the Purkinje cell layer (calbindin), and interneuron/Purkinje cells (parvalbumin) of the mature cerebella (Ce). No difference was found between the *Rsf1*^*ctrl*^ and *Rsf1*^*Nes-Cre*^ animals. Mo molecular layer; Pur Purkinje cell layer; Gr granule cell layer. **d** Western blot analysis of the mature (2-month old) brains with markers for neurons (NeuN), astrocytes (GFAP), oligodendrocytes (NG2, Olig1 and 2, and CNPase), and interneurons (calretinin, somatostatin, parvalbumin, GABA[GAD67], and calbindin)

*Rsf1* cKO mice were born at an expected ratio and had a lifespan comparable to those of control (Ctrl) animals (Figure S1e). Also the cKO animals did not show any noticeable neurological phenotypes. The overall neuroanatomical features at the microscopic level were normal, with mature neurons and astrocytes observed in the hippocampus of the *Rsf1* cKO brain (Fig. 1a). The distributions and densities of oligodendrocytes and interneurons as exemplified by parvalbumin positive neurons in the *Rsf1* cKO cerebral cortex were comparable to those in Ctrl groups (Fig. 1b). The architecture of cerebella in *Rsf1* cKO mice was also neuroanatomically intact (Fig. 1c) and these animals did not show any sign of ataxia. The protein levels of several markers for neurons, astrocytes, oligodendrocytes, and interneurons were similar between the *Rsf1* Ctrl and cKO brains (Fig. 1d). Thus, we concluded *Rsf1* is not essential for neurodevelopment. This result is in stark contrast to the *Snf2h* cKO animal model which showed several neurological defects including cerebellar abnormalities and ataxia^25^. In this animal model, the *Snf2h* gene was also selectively inactivated in the neuroprogenitor cells^25^.

### *Rsf1* is involved in apoptosis in response to DNA damage during neurodevelopment

**Fig. 2:**
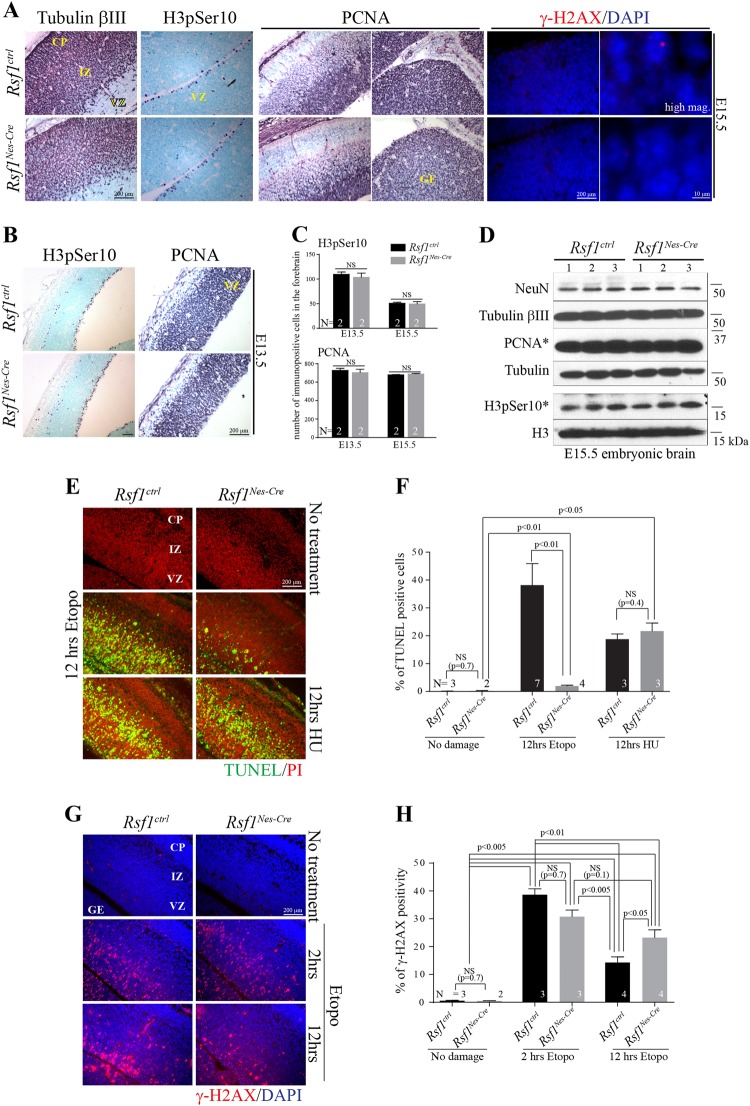
*Rsf1* deficiency prevents neural death from exogenously induced DNA damage during neurogenesis. **a** Immunohistochemical analysis of embryonic day (E) 15.5 mouse embryos with markers for neuronal differentiation (tubulin βIII), proliferation (PCNA), and neuroprogenitor cells at metaphase (H3pSer10). The overall neural development of *Rsf1*^*Nes-Cre*^ embryos was comparable with that of *Rsf1* Ctrl embryonic brain. Nuclear γ-H2AX foci were not noticeable in both *Rsf1* Ctrl and cKO embryonic brains. γ-H2AX immunoreactivity is used to detect endogenous DNA damage during neurogenesis. CP cortical plate; IZ intermediate zone; VZ ventricular zone; GE ganglionic eminence; high mag. higher magnification. **b** Immunohistochemical analysis of E13.5 mouse embryos with H3pSer10 and PCNA. **c** Quantification analysis of H3pSer10 and PCNA immunopositive cells in the ventricular zone of the developing forebrain at E13.5 and E15.5. NS not significant; *N* numbers of embryos analyzed (multiple sections of embryonic brain were obtained.) **d** Western blot analysis of several *Rsf1* Ctrl and cKO embryonic brains at E15.5. *Proliferation markers. NeuN and Tubulin βIII (neuronal differentiation and maturation), tubulin and Histone 3 were used for loading control. **e**, **f**. Examination of neural apoptosis via TUNEL (**e**) and quantification (**f**) after DNA damage induced by etoposide (Etopo) and hydroxyurea (HU) (12 h posttreatment) at E15.5. Green signals indicate TUNEL-positive staining with PI (red) counterstaining. A dramatic difference in apoptosis signal was seen in the *Rsf1*^*Nes-Cre*^ embryonic brain after etoposide treatment, yet no difference was found after HU treatment. **g**, **h** DNA damage visualized as nuclear foci formation using γ-H2AX immunoreactivity (**g**) and quantification (**h**) in the embryonic forebrains at 2 and 12 h after Etoposide (Etopo) treatment. The strong nuclear staining of γ-H2AX found in *Rsf1*^*ctrl*^ embryonic brains at 12 h time point after drug treatment is false positive. *N* numbers of embryos analyzed (multiple sections of embryonic brain were obtained)

We also looked into embryonic brain during neurogenesis, since it has been reported that RSF1 plays a role in mitosis in vitro^26^. The distributions of proliferation (phosphorylated histone 3 for metaphase and proliferating cell nuclear antigen (PCNA)) and neuronal maturation (Tubulin βIII) markers were similar in the *Rsf1* Ctrl and cKO embryonic brains (Fig. 2a, b). There was no ectopic expression of proliferating markers in the *Rsf1* cKO embryonic brains. Also, the number of proliferating cells and the protein levels of proliferation markers in the embryonic forebrains did not differ in *Rsf1* Ctrl and cKO animals (Fig. 2c, d). The normal index of proliferation and differentiation in *Rsf1* cKO embryonic brains is most likely due to the fast cell cycle and characteristic symmetric/asymmetric cell division during neurogenesis^27^.

As RSF1 was shown to be a regulatory factor for the DDR^15^, we assessed the effect of *Rsf1* deficiency on the repair capacity for endogenously induced DNA damage during neurogenesis. Endogenous DNA damage, particular strand breaks were evidently visible, only when proteins for DDR or DNA damage repair were inactivated in the developing nervous system, implying that endogenous DNA breaks naturally occur and are repaired during brain development^22,28^. DNA damage, visualized as foci of γ-H2AX immunoreactivity in the nucleus, was absent from the embryonic brains of the *Rsf1* Ctrl and cKO mice (Fig. 2a), suggesting that *Rsf1* deficiency does not affect the normal repair machinery against endogenously induced DNA damage during neurogenesis.

Next, to examine the any role of *Rsf1* in the DDR during neurogenesis, DNA strand breaks were exogenously introduced by administering either etoposide or hydroxyurea (HU) to the embryos. Both reagents induced massive amounts of apoptosis, detected by TUNEL, in the developing brain, especially in the ventricular zone where neuroprogenitors reside in (Fig. 2e, f). While *Rsf1* deficiency did not influence apoptosis caused by HU treatment, it was protective against apoptosis triggered by etoposide-induced DNA strand breaks (Fig. 2e, f), implying that *Rsf1* is one of key defenders against a certain type of DNA damage, particularly double-strand breaks, as suggested previously^15^. DNA strand breaks detected by γ-H2AX immunoreactivity were substantially high, yet similar between *Rsf1* Ctrl and cKO embryos at 2 h time point post etoposide treatment (Fig. 2g, h). This observation indicated that the etoposide treatment produced excessive DNA damage which likely surpassed the repair capability, resulting in apoptosis (Fig. 2e). However, some damaged cells in Ctrl embryonic brains were able to escape apoptosis and likely repaired DNA damage to a certain degree, as shown in reduced γ-H2AX foci, 12 h posttreatment (Fig. 2h). In contrast, significant numbers of γ-H2AX foci were still remained in *Rsf1* cKO embryonic brains at the same time point (Fig. 2h). These results suggest that *Rsf1* deficiency in the developing brain hinders both DNA damage repair and apoptosis triggered by etoposide insult.

### *RSF1* KO impairs p53 signaling and p53-induced cell death

**Fig. 3:**
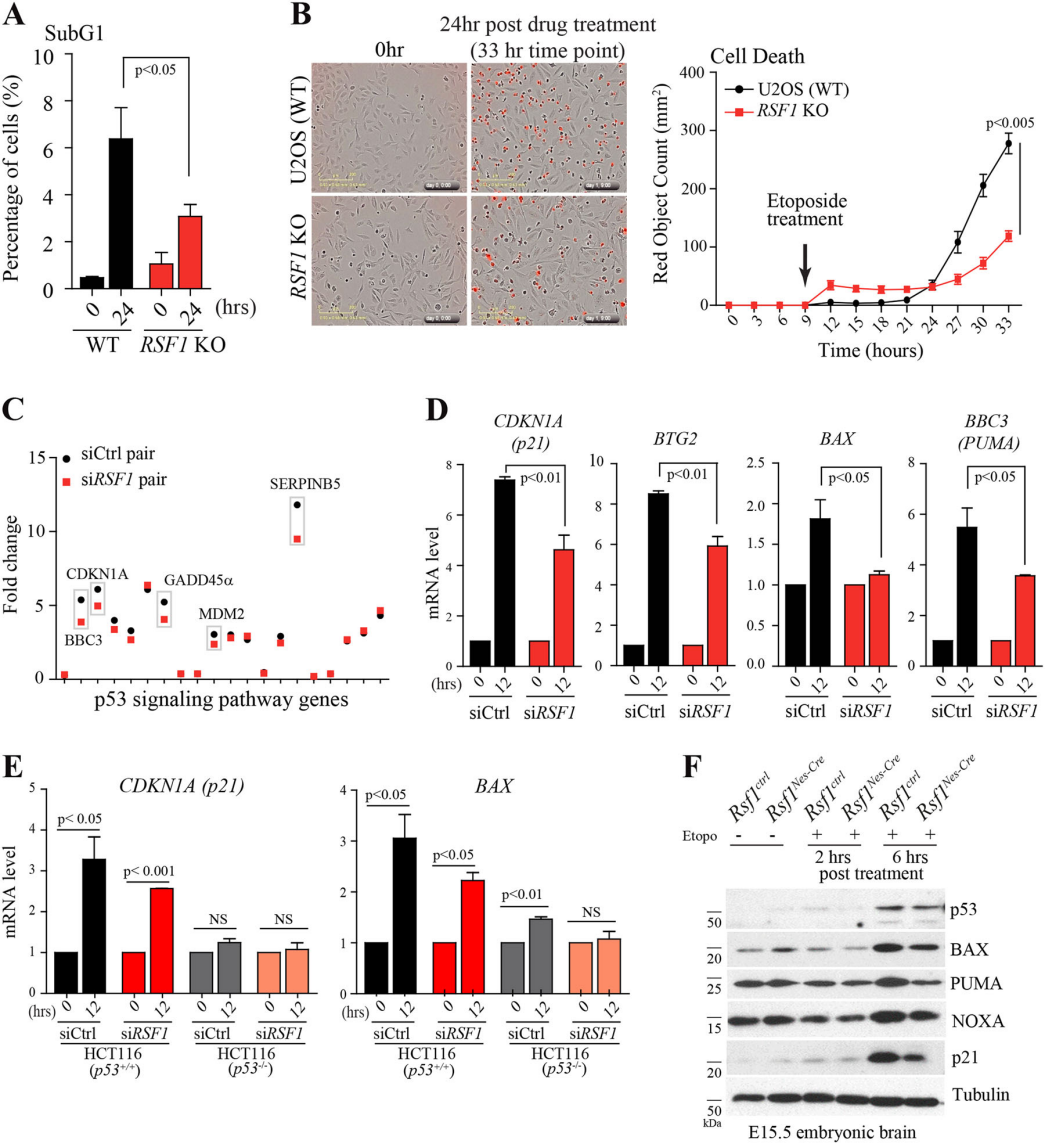
*RSF1* KO hinders p53 signaling to reduce cell death after DNA damage. **a** SubG_1_ populations of U2OS WT and *RSF1* KO cells after treatment with etoposide (68 μM) for 24 h stained with PI and counted by FACS. **b** U2OS wild-type (WT) and *RSF1* KO cells were treated with etoposide and IncuCyte Cytotox Red reagent simultaneously. Cell death indicated by red fluorescence was imaged every 3 h for 24 h after drug treatment, and counted by IncuCyte. **c** Scatter plot of fold changes of gene expression in the p53 signaling pathway in *RSF1* WT and KO cells. The same gene pair between Ctrl and *RSF1* KO cells is marked in a gray box. **d** Fold induction in the expression of p53-target genes (*CDKN1A*, *BTG2*, *BAX*, and *PUMA*) in *RSF1* WT Ctrl and deficient U2OS cells after etoposide treatment was confirmed by quantitative PCR. Gene expression induced by drug treatment was normalized to the basal level without drug treatment. **e** Fold induction in the expression of p53-target genes (*CDKN1A* and *BAX*) in HCT116 p53 proficient *(p53*^*+*/*+*^) and deficient (*p53*^*−*/*−*^) cells after etoposide treatment was confirmed by quantitative PCR. si*RSF1* was also applied. Gene expression induced by drug treatment was normalized to the basal level without drug treatment. **f** Western blot analysis of p53-target proteins (BAX, PUMA, NOXA, and p21) in the embryonic brains at E15.5 after etoposide treatment. The similar level of p53 stabilization in both Rsf1 Ctrl and cKO embryonic brains was detected after etoposide treatment

To study the mechanism by which RSF1 suppresses apoptosis further, transcription activator-like effector nucleases technology was used to generate *RSF1* KO in a human U2OS cell line. The subG_1_ population, which represents dying cells, was stained with propidium iodide (PI) and quantified by fluorescence-activated cell sorting (FACS) after treatment with etoposide for 24 h (Fig. 3a). The induction of cell death was significantly reduced in *RSF1* KO cells, comparable with in vivo results (Fig. 2e, f). Furthermore, to monitor the kinetics of apoptosis, the numbers of dead cells stained with IncuCyte Cytotox Red reagent were quantified in the images of these cells taken every 3 h. Compared with wild-type (WT) Ctrl U2OS cells, etoposide-treated *RSF1* KO cells had less red fluorescence after 24 h post etoposide treatment (Fig. 3b and Figure S2a), implying reduction in apoptosis after DNA strand breaks. Next, RNAseq was performed on *RSF1* KO and WT Ctrl cells with and without 12 h etoposide treatment to investigate differential gene expression after DNA strand breaks (Figure S2b). Similar to a previous report^29^, the majority of transcripts were upregulated under the condition of DNA damage. An ingenuity pathway analysis (IPA) revealed that the most affected genes with differential expression between *RSF1* KO and WT Ctrl cells are involved in the p53 signaling pathway (Fig. 3c and Figure S2c). We carried out several experiments to validate the results from bioinformatics analysis. The quantitative real-time PCR to measure the mRNA levels showed significant reduction of p53-target gene expressions, including *CDKN1A (p21)*, *BTG2 (BTG antiproliferation factor 2)*, *BAX (BCL2-associated X)*, *and BBC3 (BCL2-binding component 3*, *PUMA)*, in *RSF1* deficient cells compared to *RSF1* WT Ctrl cells 12 h after etoposide treatment (Fig. 3d). *p53* dependency of these target gene expressions after DNA damage was verified using HCT116 *p53*^*+*/*+*^ and *p53*^*−*/*−*^ cell lines, which no or minor induction of p53-target genes was evident in *p53* deficient cells after DNA damage regardless the status of *RSF1* (Fig. 3e, and figure S2e, S2f). This aberrant transcriptional response was not a reflection of altered p53 levels, which were maintained in *RSF1* KO cells after DNA damage and were comparable to those in WT Ctrl cells (Figure S2d and S2e). We also measured the changes of *p53*-target proteins in the E15.5 embryonic brains after etoposide treatment. p53 stabilization was similar in *Rsf1* WT and cKO embryonic brains after etoposide treatment, yet p53-target proteins including BAX, PUMA, NOXA, and p21 were less induced in *Rsf1* cKO embryonic brain compared to those of *Rsf1* WT brains after the drug treatment (Fig. 3f) similar to the in vitro analysis (Fig. 3d and Figure S2d). These data suggest that there is a connection between *RSF1* deficiency and the gene expression of p53-target genes upon DNA strand breaks.

### RSF1-dependent regulation of p53 transcriptional activity upon DNA damage

**Fig. 4:**
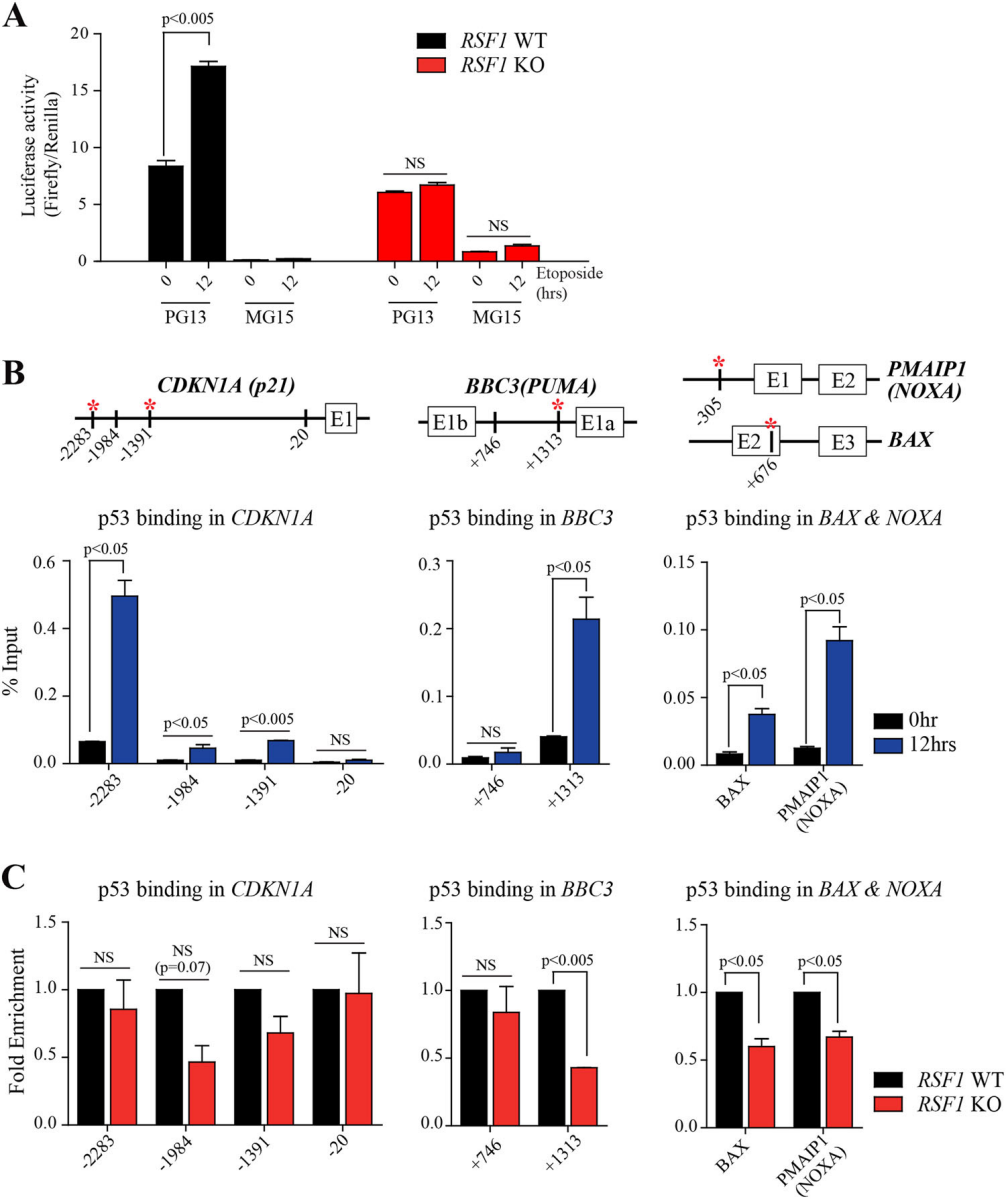
*RSF1* KO regulates binding of p53 to target gene promoters. **a** Luciferase assay to measure p53 activity for transcriptional regulation upon DNA damage. PG13 contains 13 repeats of p53 binding sites, and MG15 contains 15 repeats of the mutated binding site. NS not significant. **b** ChIP analysis of p53 at the promoters of *CDKN1A* (p21, left, with a schematic diagram of the promoter region of *CDKN1A*) and *BBC3* (PUMA, middle, with a schematic diagram of the promoter region of *BBC3*), and the p53 response elements (RE) of *BAX*, and *PMAIP1* (NOXA, right, with schematic diagrams of the p53-RE locations). The enrichment of p53 on these promoters after treatment with etoposide was calculated and represented in % input. In schematic diagrams of genes, red asterisks indicate p53 response elements in the promoters or the gene body. **c** Comparison of ChIP analysis of p53 at the promoters of *CDKN1A* and *BBC3*, and the p53 response elements (RE) of *BAX*, and *PMAIP1* between *RSF1* WT and KO cells. The level of enrichment of p53 at the promoters and p53REs after treatment with etoposide was normalized to the level before treatment in control cells

In response to DNA damage, p53 activity regulates the p53-mediated transcriptional response of its target genes. To test whether RSF1 regulates p53 activity, first we examined the activity of p53 upon DNA damage in *RSF1* WT Ctrl and KO cells. Luciferase activity with p53 binding sites (PG13) was significantly increased in *RSF1* WT Ctrl after etoposide treatment, yet the activity remained unchanged in *RSF1* KO in the same condition (Fig. 4a). The negative control with the binding site mutants (MG15) did not show any significance in luciferase activity (Fig. 4a). This result suggests that RSF1 regulates p53 transcriptional activity and its deficiency results in the aberrant transcriptional response of p53-target genes upon DNA damage.

Next, the binding of p53 to the target genes, particularly to p53 response elements (p53RE), which is a necessary step to regulate gene expression, was examined in *RSF1* KO cells. Chromatin immunoprecipitation (ChIP) was performed with p53 antibodies in *RSF1* WT Ctrl and KO cells. The fold enrichment of p53 at p53RE and non-p53RE sites in the *CDKN1A* (*p21*) and *BBC3 (PUMA)* promoter, and p53REs in *BAX* and *PMAIP1 (NOXA)* after the drug treatment was assessed in *RSF1* WT Ctrl and KO U2OS cells. p53 binding on p53RE in the target genes was significantly enriched after etoposide treatment in U2OS WT Ctrl cells (Fig. 4b). However, p53 binding to p53REs of apoptotic genes (*PUMA*, *BAX*, and *PMAIP1*) was significantly reduced in *RSF1* KO cells after DNA strand breaks (Fig. 4c), yet difference of p53 binding to *CDKN1A* was not statistically significant in this experimental setting. Thus, *RSF1* deficiency impairs the p53 activity and its binding to target gene promoters and p53RE, resulting in lower gene-expression levels, particularly related to apoptosis, upon DNA strand breaks induced by etoposide treatment.

**Fig. 5:**
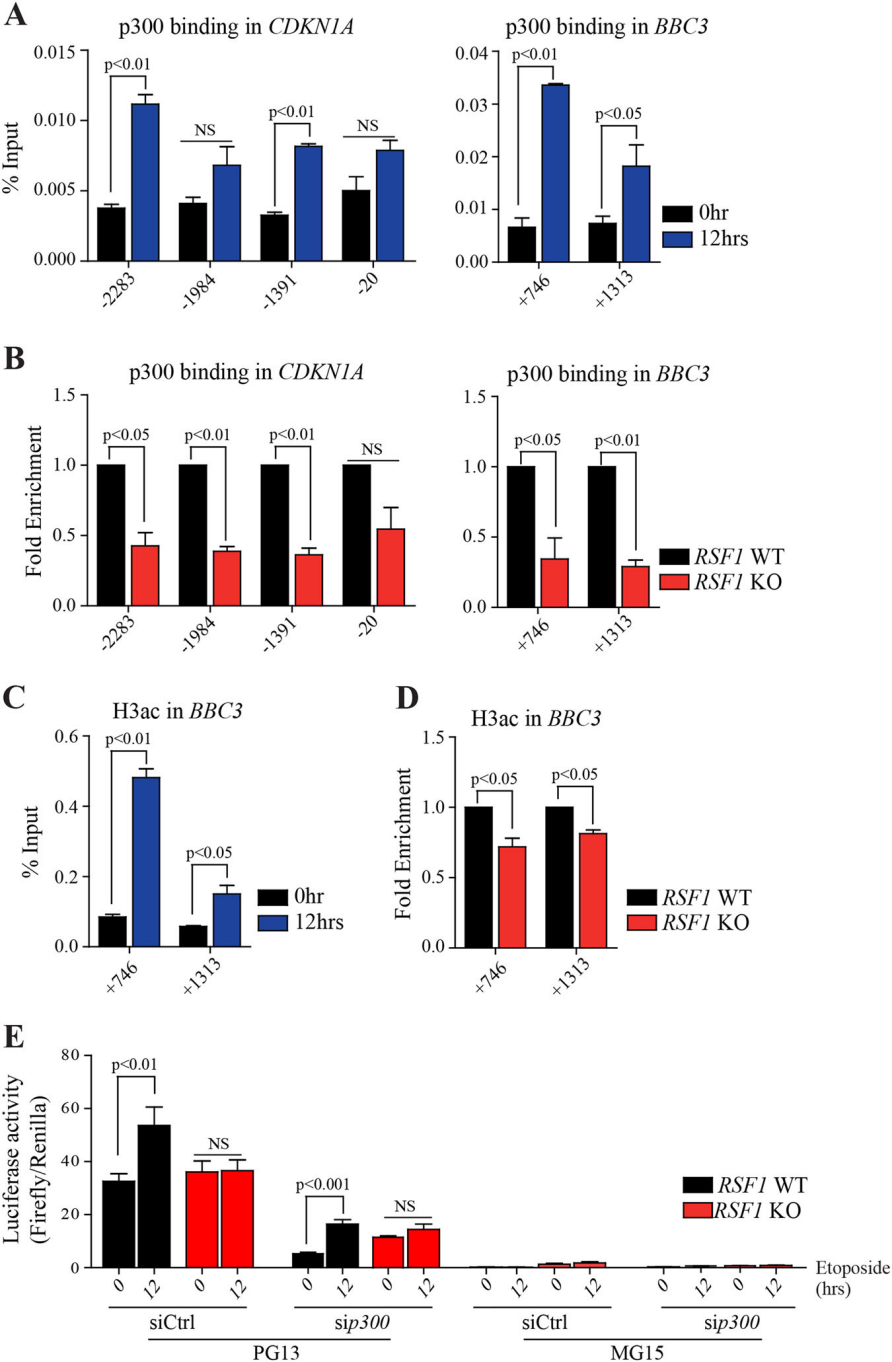
*RSF1* KO impairs p300 binding and the subsequent histone H3 acetylation on p53-target promoters. **a** ChIP analysis of histone p300 at the promoters of *CDKN1A* (p21, numbers: see Fig. 4) and *BBC3* (PUMA, middle, numbers: see Fig. 4), and p53 response elements of *BAX*, and *PMAIP1* (NOXA, the locations: see Fig. 4). The enrichment of p300 on these promoters after treatment with etoposide was calculated and represented in % input. NS not significant. **b** The level of enrichment of p300 at the promoters after treatment with etoposide in *RSF1* KO cells was normalized to the level before treatment in control cells. NS not significant. **c** ChIP analysis of histone H3 acetylation (H3ac) at the promoters of *BBC3* (PUMA). The enrichment of histone H3 acetylation on these promoters after treatment with etoposide was calculated and represented in % input. **d** The level of enrichment of histone H3 acetylation at the promoters after treatment with etoposide in *RSF1* KO cells was normalized to the level before treatment in control cells. **e** Luciferase assay to measure p53 activity for transcriptional regulation upon DNA damage. The activity of p53 for transcription was measured in the condition of *p300* or *RSF1* deficiency, as well as double deficiency of *p300* and *RSF1* after etoposide treatment. PG13 contains 13 repeats of p53 binding sites, and MG15 contains 15 repeats of the mutated binding site. NS not significant

p53 forms complexes with the histone acetyltransferase p300 for transcriptional activation, such that p300 can either acetylate histones near the target promoters, thereby relaxing the chromatin structure to promote transcription, or acetylate p53 itself, thus stabilizing the protein to regulate the transcription of p53-target genes^30^. Accordingly, ChIP experiment with p300 antibody showed the enrichment of p300 on both p53RE and non-p53RE in the promoters of p53-target genes after etoposide treatment (Fig. 5a and Figure S3a). Compared to WT cells, p300 association with promoters of p53-target genes was reduced in *RSF1* KO cells (Fig. 5b and Figure S3b). Moreover, the acetylation of histone substrates, particularly histone H3, was increased in *BBC3* promoters and enriched with p300/p53 complex after etoposide treatment in WT Ctrl cells. However, its acetylation was reduced in *RSF1* KO cells (Fig. 5c, d), indicating that the chromatin structure is not permissive for apoptotic gene expressions in *RSF1* deficiency upon DNA strand breaks. We further tested whether *p300* deficiency could affect this regulation. The expression of *p300*, which was suppressed using siRNA (Figure S3c), led to dramatic reduction of p53 activity without DNA damage, even though DNA damage-induced p53 activity in this experimental condition (Fig. 4e), suggesting that posttranslational modification of p53 is important for its activity. This upregulation after DNA damage was abolished in *RSF1* deficiency (Fig. 4e), implying the critical role of RSF1 in p53 transcriptional activity upon DNA damage. Taken all these together, the results suggest that chromatin modification by RSF1 and p300 acetyltransferase on p53 REs of its target genes is necessary for proper p53 transcriptional activity upon DNA strand breaks.

## Discussion

The structure of chromatin in higher organisms may present a hurdle toward the repair of DNA damage, as access to the sites of damage is crucial. Thus, the regulation of chromatin dynamics by chromatin-remodeling factors is requisite for proper responses to DNA damage^2^. The chromatin-remodeling factor RSF1 is involved in the DDR in vitro, particularly in the ATM-dependent checkpoint signaling pathway, and in DNA double-strand break repairs^15–17^. Considering the occurrence of endogenous DNA strand breaks during brain development^22^, the results of the current study show that *Rsf1* is dispensable for murine brain development and is not involved in the repair mechanisms for endogenously induced DNA damage during brain development. However, we could not completely rule out the possibility that RSF1 might play an important function in development of other organs. According to the IMPC website (http://www.mousephenotype.org), a germline deletion of *Rsf1* results in preweaning lethality for unknown reasons. It is possible that other binding partners of SNF2h ATPase, such as WSTF (tyrosine–protein kinase, BAZ1B) and ACF1 (bromodomain adjacent to Zinc finger domain 1A, BAZ1A), that play roles in chromatin remodeling and DDR, possibly compensate for *Rsf1* deficiency during brain development^31,32^. It was reported that deletion of *Snf2h* in the mouse results in early embryonic lethality and inactivation of *Snf2h* during neurogenesis caused cerebellar ataxia^25,33^. Nevertheless, cells in the brains of *Rsf1* cKO mice were not apoptotic after etoposide treatment but were after treatment with HU, similar to a previous demonstration of different sensitivities of *RSF1* knockdown cells to DNA double- and single-strand breaks^15^.

The impaired cell death response with *RSF1* deficiency following excessive DNA strand breaks was confirmed by in vitro and in vivo analyses, which identified the reduced transcription of p53-target genes as a potential mechanism. The reduction in target gene expression after DNA damage was associated with a loss of p53 or p300 binding at the gene promoters and p53REs. Moreover, the acetylation of histones was reduced at these promoter regions after induction of DDR in *RSF1* deficiency. This reduction in binding was not a result of reduced p53 protein levels, which the protein was unchanged and comparably stabilized after DNA damage in mouse brain tissues and human *RSF1* KO cells. However, the posttranslational modification of p53 by p300 as a cause of reduced target gene expression cannot be ruled out, as the acetylation of K120 and C-terminal lysines of p53 is known to regulate the transcription of pro-apoptotic genes^30,34^. Thus, further study of p53 acetylation and phosphorylation in *RSF1* KO cells after DNA damage is warranted. Furthermore, RSF1 might directly mediate the interaction of p53 with p300 to modify the chromatin structure and activate the transcription of the target genes. As the RSF complex was initially purified with the FACT complex and identified as a transcriptional activator, it is also possible that the RSF1/SNF2h complex regulates histone modifications of H3/H4 and recruitment of p300 to specific promoters^3,4^.

**Fig. 6:**
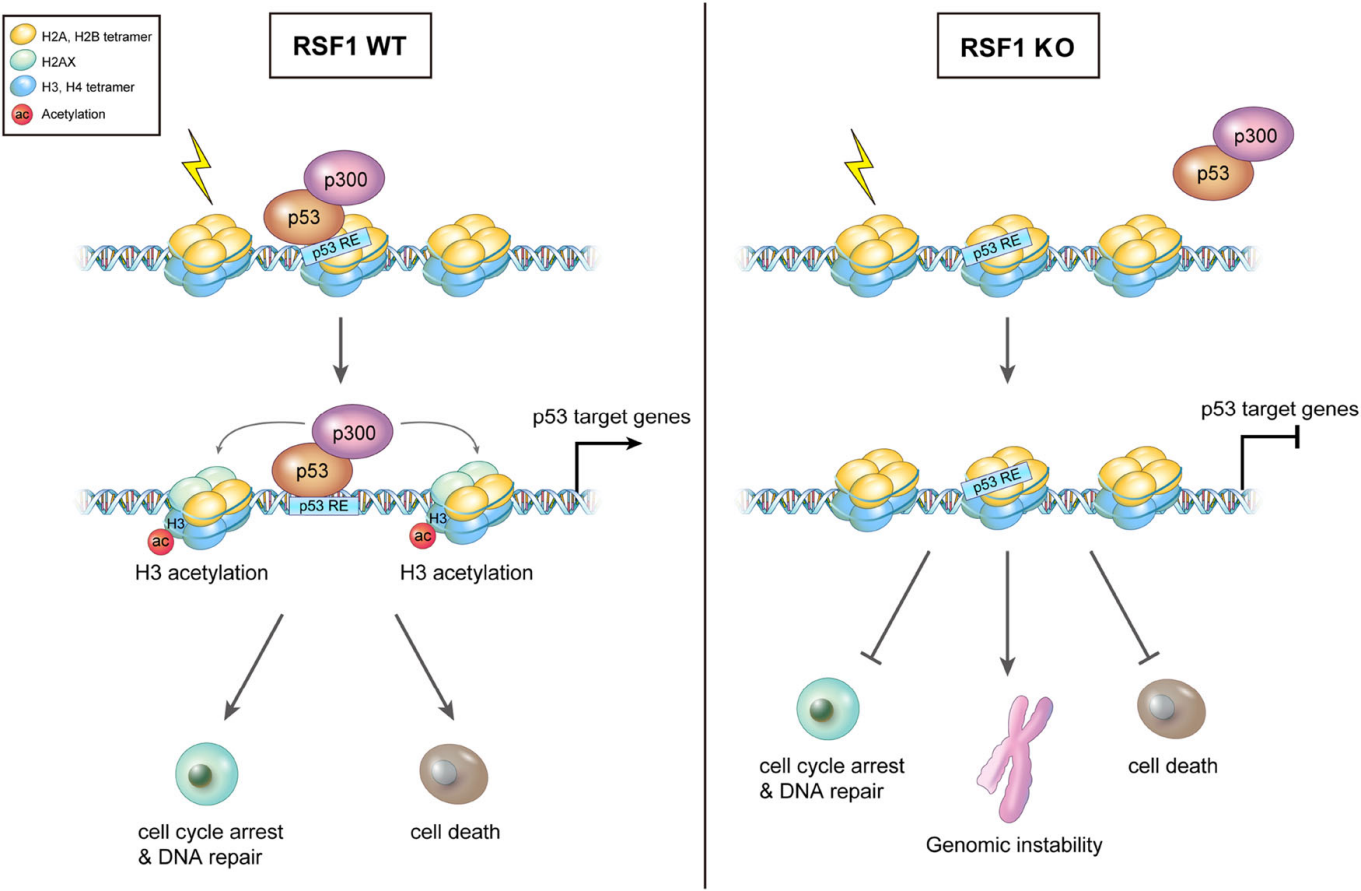
A graphic summary of the findings. In *RSF1* proficiency, p53/p300 complex stably associates with promoters (p53 RE; reponse element) of p53-target genes, which leads to the subsequent histone H3 acetylation on the promoters to induce gene expression upon DNA damage. However, *RSF1* inactivation blocks the stable association of p53/p300 complex on the promoters of p53-target genes and suppresses their expressions, especially apoptotic genes. Consequently, this condition leads to escape from cell death, resulting in genomic instability

In summary (Fig. 6), the current study shows that a loss of RSF1 impairs apoptosis and p53-dependent gene expression, particularly of genes related to cell death, after etoposide treatment in vivo and in vitro. The reduction in p53-induced gene expression was associated with a loss of p53/p300 binding at the gene promotors accompanied by altered histone modifications, likely resulting in altered chromatin structure and genomic instability.

## Materials and methods

### Animals

The animal model harboring a floxed exon 4 of the *Rsf1* gene [*Rsf1*^*tm1c(EUCOMM)*^] after breeding with a *Flp* animal line to remove the *LacZ*/*Neo* cassette flanked by the *Frt* sites (S1b) was obtained from the Toronto Centre for Phenogenomics (a facility of Mount Sinai Hospital & the Hospital for Sick Children, Canada), which is a member of the international mouse phenotyping consortium (IMPC). Up to date, the IMPC website (http://www.mousephenotype.org/data/genes/MGI:2682305) indicates that the *Rsf1*^*tm1b(EUCOMM)*^ allele, which is a reporter-tagged deletion allele, results in preweaning lethality. To induce *Rsf1* inactivation during neurodevelopment, *Rsf1*^*LoxP*/*LoxP*^ animals were crossed with *Nestin-Cre* [B6.Cg-Tg(Nes-cre)1Kln/J, stock no. 003771] animals to generate *Rsf1*^*LoxP*/*LoxP*^*;Nestin-Cre* (*Rsf1*^*Nes-Cre*^ or *Rsf1* cKO) animals. There were no differences between male and female *Rsf1* cKO mice; thus analyses were performed regardless of sex. The *Rsf1*^*LoxP*/*+*^*;Nestin-Cre* animals did not show any defects and were used as the controls. The genotypes of *Rsf1* animals were determined by routine PCR using the following primers. As shown in Figure S1b, to detect the floxed allele, Set1 primers were used [P1: 5′-TTGGAGAGATTTCGGGTAAACTTAC (forward) and P2: 5′-CAATACATGAGGCCATCTTTGTCTC (reverse)] with 34 cycles of amplification at 94 °C for 30 s, 60 °C for 45 s, and 72 °C for 1 min, which produces a 667 bp fragment for the WT allele and an 808 bp fragment for the floxed allele. To detect the targeted allele, Set2 primers were used [P3: 5′-GCGCAACGCAATTAATGATAAC (forward) and P4: 5′-ACCAGGTAAGAGTTCATGTCAAAGCAGC (reverse)] with 34 cycles of amplification at 94 °C for 30 s, 60 °C for 30 s, and 72 °C for 1 min, which produces a 437 bp fragment. The PCR for *Cre* recombinase was detected using the following primers and PCR conditions: (Cre-3: 5′-CTGCCACGACCAATGACAGC and Cre-4: 5′-ACCTGCGGTGCTAACCAGCG) with 35 cycles of amplification at 94 °C for 30 s, 60 °C for 45 s, and 72 °C for 45 s.

The presence of a vaginal plug indicated embryonic day 0.5 (E0.5), and the day of birth was designated as postnatal day 0 (P0). To induce DNA damage, etoposide (4 mg/kg) or HU (400 mg/kg) was administrated intraperitoneally to pregnant mice at E15.0, and the embryos were collected at the indicated time points. All animals were housed in the Laboratory Animal Research Center of Ajou University Medical Center and maintained in accordance with the guidelines of the Institutional Animal Care and Use Committee. All procedures for animal use were approved by the ethics committee at the center.

### Reverse transcription and quantitative real-time PCR

To measure effective deletion of floxed exon 4 in the *Rsf1* gene, quantitative real-time PCR was applied. Genomic DNA was purified from tissue samples with a phenol/chloroform solution and used for quantitative real-time PCR on a Rotor-Gene Q instrument (Qiagen) with a Rotor-Gene SYBR green PCR kit (Qiagen) and the following primers. The floxed exon 4 of *Rsf1* was amplified with primers: 5′-GGTACCAGTTGGATCAAGACCATA (forward) and 5′-ACAATGCATTTCCAAGAAGAGCCA (reverse); the intact exon 6 was amplified with primers: 5′-GGACATGCCTCTCGAACCTT (forward) and 5′-CTTCTGGGGGCCTTCTCTTC (reverse). The results of real-time PCR were normalized to those for β-actin (forward primer: 5′-CAGCAAGCAGGAGTACGATGAG; reverse primer: 5′-CAGTAACAGTCCGCCTAGAAGCA). The deletion efficacy by *Cre* recombinase was calculated as the ratio of exons 4 and 6.

For quantitative reverse transcription real-time PCR to confirm RNAseq analysis, 2 μg of total RNA isolated from several human cell lines as described below was used for cDNA synthesis with amfiRivert cDNA synthesis platinum master mix (GenDEPOT). Quantitative PCR was performed on a Rotor-Gene Q system (Qiagen) using Maxima SYBR Green qPCR master mix (Thermo) with the indicated primers (Table S1).

### Cloning of mouse *Rsf1* for microirradiation

The cloning of human *RSF1* was described previously^15^. Murine *Rsf1* was amplified from mouse brain cDNA via routine PCR, and the PCR product was cloned into a GFP-tagged vector using the Gateway cloning system (Invitrogen). For DNA damage analysis, U2OS cells were transfected with either human *RSF1-GFP* or mouse *Rsf1-GFP*. When the cells reached ~80% confluency, BrdU (final concentration, 10 μM) was added to the culture for 30 h prior to microirradiation, which was conducted with a 405 nm laser (32 repetitive insults/s for 3 s) to induce DNA strand breaks. An accumulation of GFP fluorescence at the sites of DNA damage was observed under an A1 confocal microscope (Nikon) equipped with a temperature- and CO_2_-controlled chamber.

### Cell lines and luciferase assay

For in vitro study, human cell lines [HCT116 (*p53*^*+*/*+*^), HCT116 (*p53*^*−*/*−*^), U2OS] *RSF1* proficient and deficient cell line were used for the analysis. siRNA sequence for *p300* was 5′-AACCCCUCCUCUUCAGCACCA. si*RSF1* was previously reported^15^. Luciferase assays were performed in human cell lines using PG13-luc, containing 13 copies of the p53 binding consensus sequence (PG13), and MG15-luc, containing 15 copies of mutated p53 binding sequence (MG15), reporter plasmids (Addgene). Cells were transfected with the reporter plasmids and siRNA using Lipofectamine 2000 (Invitrogen) and incubated for 48 h. Etoposide was treated for 12 h before cells were harvested. Luciferase activity was measured using a Dual-Luciferase Reporter Assay System (Promega). Firefly luciferase activity was normalized by Renilla luciferase activity, and statistically analyzed using Prism software (GraphPad).

### Western blot analysis

Mature and embryonic brain samples were microdissected from 2 to 3 *Rsf1* Ctrl or cKO animals and snap-frozen in liquid nitrogen. Western blotting was performed as described before with a slight modification^15^. Mouse brain tissues, *RSF1* proficient and deficient human cell lines were prepared in a lysis buffer containing 50 mM Tris (pH 7.5), 150 mM NaCl, 50 mM NaF, 0.2% NP-40, 1% Tween-20, 1 mM dithiothreitol, protease inhibitor (Sigma), and phosphatase inhibitor (Sigma). Protein amounts were quantified using Bio-Rad protein assay reagent. The antibodies for Western blot analyses were against NeuN (1:1000, mouse; Millipore), Tubulin βIII (Tuj1, 1:3000, mouse, BabCo), glial fibrillary acidic protein [(GFAP) 1:2000, rabbit; Cell Signaling Technology, NG2 (1:500, rabbit; Millipore), Olig1 (1:1000, rabbit; LifeSpan Biosciences), Olig2 (1:1000, mouse; Millipore), cyclic-nucleotide phosphodiesterase (CNPase) 1:2000, mouse; Sigma, calretinin (1:10,000, rabbit; Millipore), somatostatin (1:1000, rabbit; Abcam), parvalbumin (1:2500, rabbit; Abcam), glutamate decarboxylase] [(GAD67) 1:5000, rabbit; OriGene, calbindin (1:6000, mouse; Sigma)], proliferating cell nuclear antigen [(PCNA) 1:1000, mouse; Santa Cruz Biotechnology], phosphorylated histone 3 serine 10 (H3pSer10, 1:1000, rabbit, Cell signaling), histone 3 (H3, 1:5000, rabbit, Abcam), RSF1 (1:2000, rabbit; Abcam), p53 (1:1000, mouse; Santa Cruz Biotechnology and 1:1000, rabbit, Cell signaling), p21 (CDKN1A, 1:1000, rabbit, Santa Cruz Biotechnology and 1:1000, rabbit, Abcam), BBC3 (Puma, 1:2000, rabbit, Abcam), BAX (1:2000, rabbit, Abcam), Noxa (Pmaip1, 1:2000, mouse, Abcam), p300 (1:1000, Rabbit, Santa Cruz Biotechnology) and GAPDH (1:2000, rabbit; Santa Cruz Biotechnology). The equal loading of samples was confirmed by quantifying bands for anti-tubulin (1:40,000, mouse; a gift from Dr. Sang Gyu Park) and Ponceau staining.

### Histopathological analysis

Histopathological analyses were performed as described below. Briefly, embryos and adult mouse brain were fixed in 4% formaldehyde in phosphate-buffered saline (PBS), cryoprotected in 30% sucrose PBS solution, and cryosectioned (10 µm thick sagittal sections) with an MEV cryostat (SLEE Medical GmbH, Mainz, Germany). Sections were incubated with antibodies overnight after quenching endogenous peroxidase with 0.6% hydrogen peroxide for colorimetric signals. Immunopositivity was visualized with the VIP substrate kit (Vector Laboratories) with biotinylated secondary antibodies and avidin DH-biotinylated horseradish peroxidase-H complex (Vector Laboratories) or with Cy3-conjugated secondary antibodies (Jackson ImmunoResearch Laboratories). Methyl green (0.1% solution) was used for counterstaining followed by mounting with DPX (Sigma) or DAPI/propidium iodide (PI) containing mounting medium (Vector Laboratories). Citric acid-based antigen retrieval was applied when necessary. The antibodies were against tubulin βIII (Tuj1, 1:1000, mouse; BabCo), GFAP (1:500, mouse; Sigma), NeuN (1:500, mouse, Millipore), CNPase (1:2000, mouse; Sigma), calbindin (1:2000, mouse; Sigma), parvalbumin (1:1000, rabbit; Abcam), proliferating cell nuclear antigen [(PCNA) 1:500, mouse; Santa Cruz Biotechnology], phosphorylated histone 3 serine 10 (H3pSer10, 1:5000, rabbit, Cell signaling) and γ-H2AX (1:200, rabbit; Cell Signaling Technology). Apoptosis was measured with an Apoptag fluorescein in situ kit (Millipore).

### RNA isolation and RNAseq

Total RNA was isolated from indicated human cell lines with an RNeasy Plus Mini kit (Qiagen). For the RNAseq analysis, the quality of RNA was checked using Bioanalyzer RNA ChIP (Agilent Technologies). An mRNA library was prepared with a Truseq stranded mRNA kit (Illumina), and RNAseq was performed using NextSeq 500 (Illumina). To analyze RNA sequences, raw reads were preprocessed by base trimming and mapped on the reference sequence, STAR. The counted reads were quantified and analyzed by differentially expressed gene analysis and functional annotation was analyzed by DAVID and IPA. To compare the level of transcripts in control and *RSF1* depleted cells, the transcription level after DNA damage was normalized by the transcription level before DNA damage, which represents the fold change of transcripts in each indicated siRNA.

### Cell-cycle analysis

U2OS *RSF1* WT and KO cells were harvested after treatment with etoposide (60 μM) for 24 h and fixed with 80% ethanol overnight at 20 °C. The fixed samples were washed with PBS three times and stained with PI for analysis by FACS.

### Chromatin immunoprecipitation (ChIP)

Indicated human cells were harvested after treatment with etoposide (30 μM) for 12 h and cross-linked with 1% formaldehyde for 20 min. Cells were lysed with SDS lysis buffer [1% SDS, 10 mM EDTA, 50 mM Tris (pH 8.1)] supplemented with protease and phosphatase inhibitor cocktail (Thermo) for 10 min on ice. Cell lysates were sonicated with a Bioruptor (Diagenode) and centrifuged at 13,000 rpm for 15 min. The supernatants were collected and diluted for overnight incubation with primary antibody. Then, 20 μl of protein A agarose/salmon sperm DNA (Millipore) was added to each sample and incubated with rotation at 4 °C. After a 1 h incubation, the beads were washed with low-salt immune complex wash buffer [0.1% SDS, 1% Triton X-100, 2 mM EDTA, 20 mM Tris-HCl (pH 8.1), 150 mM NaCl], high-salt immune complex wash buffer [0.1% SDS, 1% Triton X-100, 2 mM EDTA, 20 mM Tris-HCl (pH 8.1), 500 mM NaCl], LiCl immune complex wash buffer [0.25 M LiCl, 1% IGEPAL-CA630, 1% deoxycholic acid (sodium salt), 1 mM EDTA, 10 mM Tris (pH 8.1)], and TE buffer (10 mM Tris-HCl, 1 mM EDTA, pH 8.0). The immune complexes were eluted in elution buffer (1% SDS, 0.1 M NaHCO_3_) for 30 min at room temperature. The eluates were incubated with 10 μl of 5 M NaCl to reverse histone–DNA cross-links by heating at 65 °C for 4 h, followed by incubation with proteinase K at 45 °C for 1 h. DNA was purified with a Nucleospin PCR clean-up kit (Macherey-Nagel) and processed for quantitative PCR. The antibodies used in ChIP assays were against p53 (Santa Cruz Biotechnology), p300 (Santa Cruz Biotechnology), and acetylated H3 (Millipore). The primers used for this analysis are described in Table S2^35,36^.

### Image and statistical analyses

Images from a B600TiFL microscope (Optika) were captured with a DFC130 digital camera (Leica), processed using Photoshop software (Adobe), and analyzed with the measuring function in ImageJ (NIH). Areas of TUNEL-positive green signals (0.27 mm^2^) in the developing forebrains were measured for green and red signals, and then the ratio of the apoptosis signal and total area was calculated. Also H3pSer10 and PCNA immunopositive cells (0.27 mm^2^) were counted in multiple sections of embryonic brains. For DNA damage, the ratio of the γ-H2AX foci area (red in 150 μm^2^) and the total nucleus area (blue) was calculated. Multiple slides (apoptosis) and several sites in each section in multiple slides (γ-H2AX) were measured, and the number of examined embryos was as indicated in Fig. 2. For cell death analysis in vitro, images from IncuCyte were captured every 3 h for 24 h after drug treatment at 9 h time point (Fig. 3b) and IncuCyte Cytotox Red reagent was automatically counted by IncuCyte. U2OS WT and *RSF1* KO cells were treated with IncuCyte Cytotox Red reagent and etoposide simultaneously. All statistical analyses were performed using Prism software (GraphPad). A *P* value of <0.05 was considered significant.

## Electronic supplementary material

Suppl. information

Suppl. Figure 1

Suppl. Figure 2

Suppl. Figure 3
