# Supplementary material for: Chromatin-remodeling factor, RSF1, controls p53-mediated transcription in apoptosis upon DNA strand breaks
Source: Cell Death Dis. 2018 Oct 22;9(11):1079. doi: 10.1038/s41419-018-1128-2 (PMC6197202; doi:10.1038/s41419-018-1128-2)
Supplement: Supplementary file 1 — Suppl. information [file 41419_2018_1128_MOESM1_ESM.docx]

**[Cell Death & Disease] – [Revision]**

Wednesday, October 3, 2018

**Title: Chromatin remodeling factor, RSF1, controls p53 mediated transcription in apoptosis upon DNA strand breaks**.

**Authors:** Sunwoo Min^1,3,*^, Keeeun Kim^1,4,*^, Seong-Gwang Kim^1,3,4^, Hyeseong Cho^1,2,3,4^ and Youngsoo Lee^1,2,4^

**Affiliation: ^1^**Genomic Instability Research Center, **^2^**Genome Stability Institute, and **^3^**Department of Biochemistry & Molecular Biology, Ajou University School of Medicine, Suwon, 16499, Korea, **^4^**Department of Biomedical Sciences, The Graduate School of Ajou University, Suwon, 16499, Korea

**Address correspondence to:**

**Youngsoo Lee**, Genomic Instability Research Center, Ajou University School of Medicine, Suwon, 16499, Korea, Phone; 82-31-219-7805, Fax; 82-31-219-7802, E-mail; ysoolee@ajou.ac.kr

* S.M. and K.K. contributed equally to this work.

Running (short) title: RSF1-dependent p53 signaling in DDR

Keywords: DNA damage, apoptosis, RSF1, p53

6 figures, 3 Supplementary figures, and 2 Supplementary Tables

**SUPPLEMENTARY INFORMATION**

**Supplementary Figure 1. New *Rsf1* cKO animal model.**

**a.** Representative pictures of RSF1 accumulation at the sites of DNA strand breaks induced by micro-irradiation. U2OS cells were transfected with either a human *RSF1(hRSF1)-GFP* or mouse *Rsf1(mRsf1)*-GFP construct. Red arrows indicate the site of DNA damage and the accumulation of RSF1.

**b.** Upper left: A schematic diagram showing domains in mouse RSF1 protein. The targeted part of the floxed mouse *Rsf1*, which spans two WHIM domains, is indicated by the red line to generate a conditional knockout mouse model. Lower left: routine PCR products for genotyping with primers as indicated in the gene construct (right panel: P1/P2 and P3/P4). The genotypes of animals were determined by the floxed *Rsf1* allele products with or without *Nes-Cre* expression. The PCR products with Set1 primers indicate whether animals have the floxed *Rsf1* allele. And with Set2 primers, excision of the exon 4 in the floxed *Rsf1* gene by *Cre* recombinase is detected. Right: the engineered gene construct of the *Rsf1* gene (exon 4 was floxed) and strategy for gene deletion by *Flp* recombinase to target *FRT* sites and *Cre* recombinase to target *LoxP* sites. DDT, DNA-binding homeobox-containing proteins and the different transcription and chromatin remodeling factors domain; W1/W2, WHIM domain; PHD, plant finger homeodomain; E, exon; FRT, short flippase recognition target; Neo, neomycin-resistant cassette; Flp, flippase; loxP, locus of X-over P1; P, primer.

**c.** Real-time PCR analysis to confirm selective gene targeting by *Cre* recombinase expression driven by the *Nestin* promotor; the efficiency of targeting was calculated by the ratio of exons 4 (targeted) and 6 (intact). The targeted portion of *Rsf1* is absent only in the brains, including the cerebral cortex, cerebellum, diencephalon, and hippocampus, of the *Rsf1^Nes-cre^* animals in comparison with those of the *Rsf1^Ctrl^* animals.

**d.** Western blot analysis indicates that RSF1 protein is absent in the *Rsf1^Nes-Cre^* brain. The red arrow head indicates RSF1 immunopositive bands. * indicates non-specific bands. mRSF1, mouse RSF1 protein

**e.** Kaplan–Meier survival curve of Control and *Rsf1* cKO animals. These animals were observed for their normal life span. There was no difference in survival between Control and *Rsf1* cKO animals [log-rank (Mantel-Cox) test, p = 0.96].

**Supplementary Figure 2. *RSF1* KO reveals defects in the p53 signaling pathway.**

**a.** Cells with red fluorescence in control and *RSF1* KO cells were counted every three hours up to 60 hrs to measure cell death, and normalized by the number of cells in DIC images. The cells were treated with etoposide at 9 hr time point (main figure 2b).

**b.** An experimental scheme for RNAseq. Control and *RSF1* KO cells were divided into two groups: one was without DNA damage induction and DNA strand breaks were introduced by etoposide treatment in another group. Theses samples were subject to RNAseq analysis.

**c.** Results from RNAseq were analyzed via IPA (Ingenuity Pathway Analysis). The p53 signaling pathway showed significant reduction in *RSF1* pair comparison compared with the fold change in control cells. The fold change was calculated by normalizing the level of gene expression after treatment with etoposide for 12 hrs to the level of gene expression before treatment.

**d.** Protein levels of p53, along with p21 and RSF1 were analyzed by Western blotting. p53 stabilization in RSF1 KO cells was comparable to that of RSF1 WT Ctrl cells after DNA damage.

**e.** Confirmation of p53 status in HCT116 p53 proficient (*p53^+/+^*) and deficient (*p53^-/-^*) cells by Western blot analysis.

**f.** Fold induction in the expression of p53 target genes (*NOXA*) in HCT116 *(p53^+/+^*) and HCT116 (*p53^-/-^*) cells after etoposide treatment was confirmed by quantitative PCR. si*RSF1* was also applied. Gene expression induced by drug treatment was normalized to the basal level without drug treatment. Although the statistical analysis indicated the comparisons were not significant (NS), the tendency of gene expression pattern was similar to those of *p21* and *BAX*.

**Supplementary Figure 3. *RSF1* KO reduces the binding of p53 to promoters of apoptosis-regulating genes such as *BAX* and *PMAIP1*.**

**a-b.** ChIP analysis of p300 enrichment on the p53 response elements on *BAX* and NOXA **(A)** in WT Ctrl cells after the drug treatment. Although the statistical analysis indicated the comparisons were not significant (NS), the tendency of gene expression pattern was similar to those of *p21* and *BBC3* after DNA damage. The comparison of p300 binding to the p53 response elements on *BAX* and *NOXA* in WT Ctrl and *RSF1* KO cells (**B)** after etoposide treatment. U2OS WT and *RSF1* KO cells were treated with etoposide for 12 h prior to ChIP analysis with a p300 antibody.

**c.** Confirmation of the protein level of p300 by siRNA by Western blot.

**Supplementary Table 1. qPCR primers.**

| Gene target | Primer sequence |
| --- | --- |
| *CDKN1A* (*P21*) | FW: TTG TAC CCT TGT GCC TCG CT  RV: TTG GAG AAG ATC AGC CGG C |
| *NOXA* | FW: GGA GAT GCC TGG GAA GAA G  RV: CCT GAG TTG AGT AGC ACA CTC G |
| *BTG2* | FW: GAA AAG CCG TCC AAG GGC  RV: CTT GTG GTT GAT GCG AAT GC |
| *BAX* | FW: AAG AAG CTG AGC GAG TGT  RV: GGC GGC AAT CAT CCT CTG |
| *BBC3* (*PUMA*) | FW: GCA GGC ACC TAA TTG GGC T  RV: ATC ATG GGA CTC CTG CCC TTA |
| *GAPDH* | FW: CTC TGC TCC TCC TGT TCG AC  RV: ACG ACC AAA TCC GTT GAC TC |
| *ACTB* | FW: AGC CGG GCT CTT GCC AAT  RV: AGT TAG CGC CCA AAG GAC CA |

FW, forward; RV, reverse.

**Supplementary Table 2. ChIP-qPCR primers.**

| Primer name | Sequence |
| --- | --- |
| p21_-20 | FW: AGC TGC GCC AGC TGA GG  RV: CAC AAG GAA CTG ACT TCG GCA |
| p21_-1391 | FW: CTG TCC TCC CCG AGG TCA  RV: ACA TCT CAG GCT GCT CAG AGT CT |
| p21_-1984 | FW: CAG AAG TCC TCC CTT AGA GTG TGT CT  RV: GCA ACC ATG CAC TTG AAT GTG TA |
| p21_-2283 | FW: AGC AGG CTG TGG CTC TGA TT  RV: CAA AAT AGC CAC CAG CCT CTT CT |
| PUMA_+1313 | FW: TCA GTG TGT GTG TCC GAC TGT C  RV: GGC AGG GCC TAG CCC A |
| PUMA_+746 | FW: GTC GGG CGA ATG TCA CTT TC  RV: CCT GGA TAC ACG GCC AAA TC |
| BAX_p53RE | FW: AGA TCA TGA AGA CAG GGG CCC TTT  RV: TGG AGT GAG GGT GCA GAA TCA GAA |
| NOXA_p53RE | FW: CAG CGT TTG CAG ATG GTC AA  RV: CCC CGA AAT TAC TTC CTT ACA AAA |
