# Supplementary figures and images for: Chromatin-remodeling factor, RSF1, controls p53-mediated transcription in apoptosis upon DNA strand breaks
Source: Cell Death Dis. 2018 Oct 22;9(11):1079. doi: 10.1038/s41419-018-1128-2 (PMC6197202; doi:10.1038/s41419-018-1128-2)

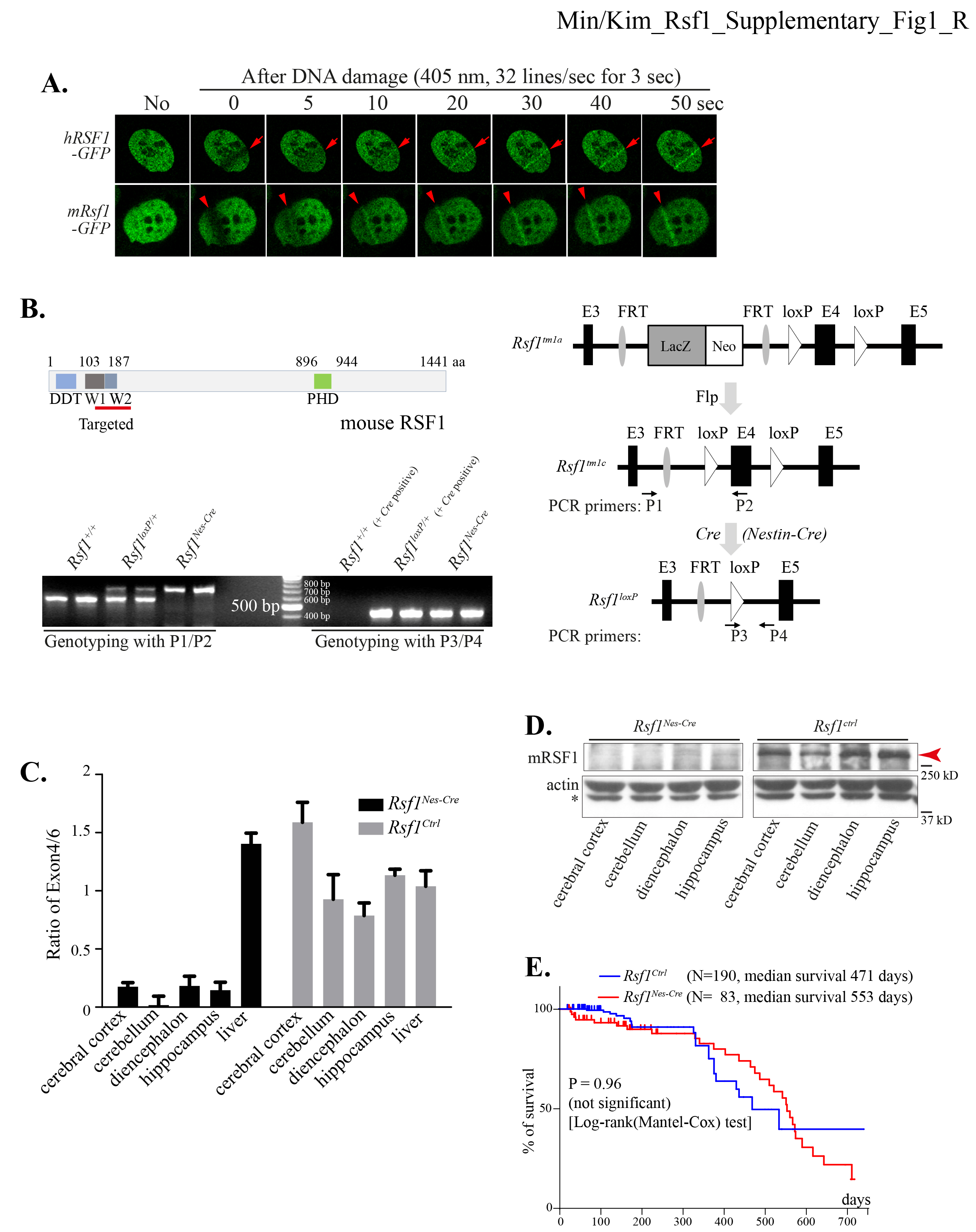

Supplement: Supplementary file 2 — Suppl. Figure 1 [file 41419_2018_1128_MOESM2_ESM.jpg]

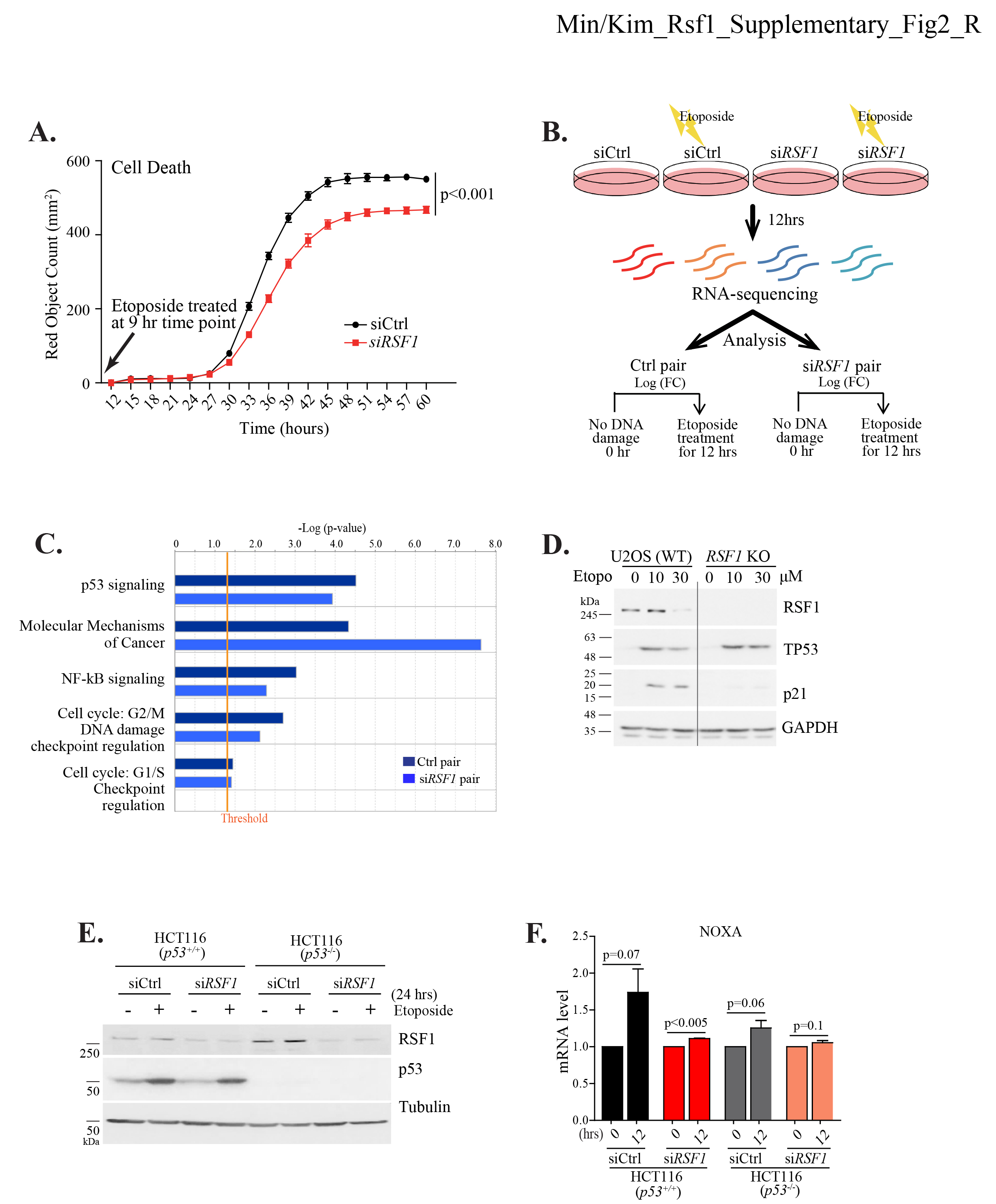

Supplement: Supplementary file 3 — Suppl. Figure 2 [file 41419_2018_1128_MOESM3_ESM.jpg]

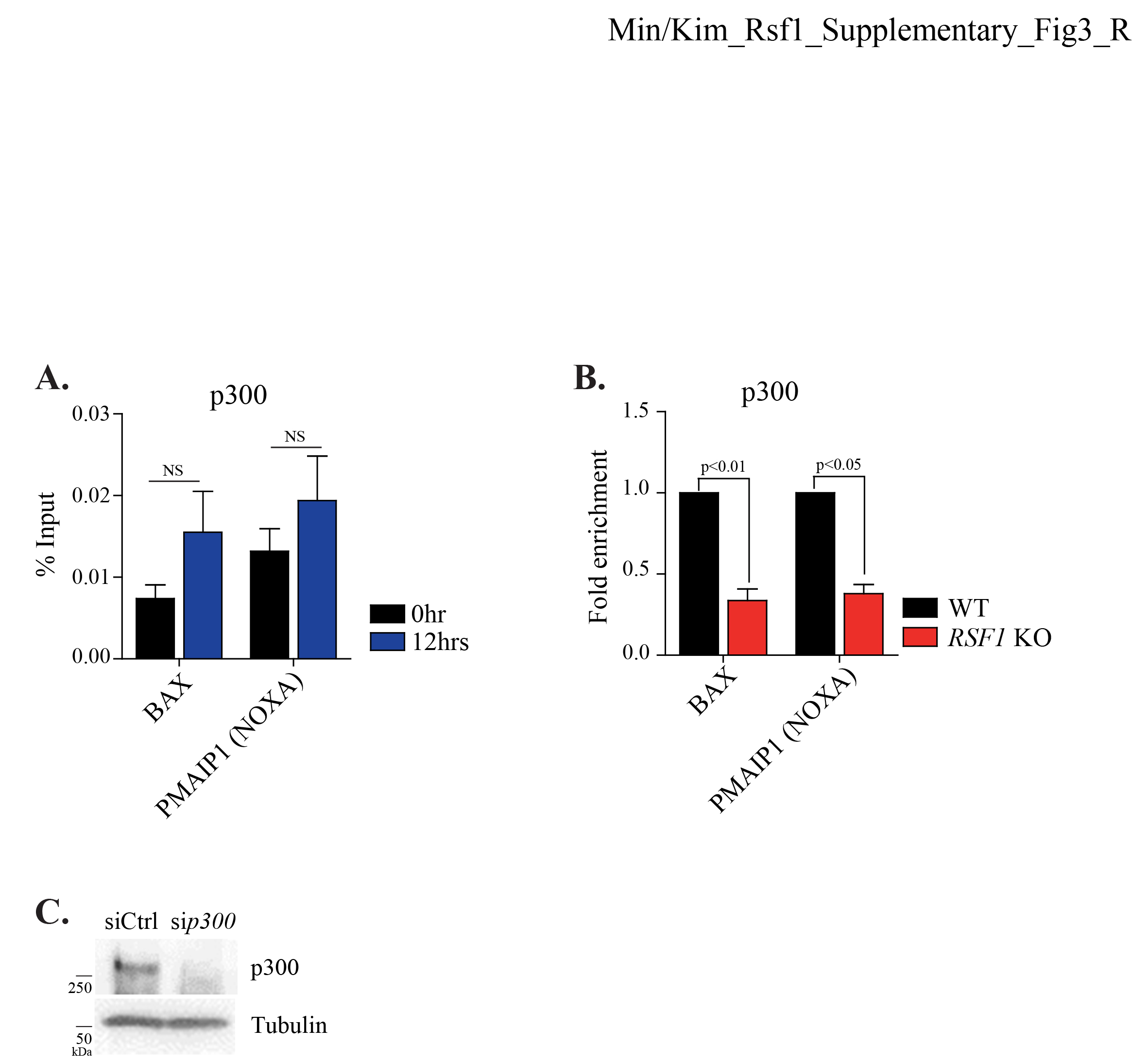

Supplement: Supplementary file 4 — Suppl. Figure 3 [file 41419_2018_1128_MOESM4_ESM.jpg]
